# Orally administered low-molecular weight agaro-oligosaccharides are absorbed into the plasma of healthy humans

**DOI:** 10.3389/fnut.2023.1273328

**Published:** 2023-09-25

**Authors:** Ikuya Shirai, Yu Iwasaki, Koji Karasawa, Yasutaka Shigemura, Shigeru Katayama

**Affiliations:** ^1^Department of Science and Technology, Graduate School of Medicine, Science and Technology, Shinshu University, Nagano, Japan; ^2^Ina Food Industry Co., Ltd., Nagano, Japan; ^3^Faculty of Domestic Science, Tokyo Kasei University, Tokyo, Japan; ^4^Department of Biomolecular Innovation, Institute for Biomedical Sciences, Shinshu University, Nagano, Japan

**Keywords:** agaro-oligosaccharide, agarobiose, human plasma, liquid chromatography/mass spectrometry, pharmacokinetics

## Abstract

Agaro-oligosaccharides (AOSs) are known to have biological activities, such as anti-inflammatory, anti-tumor, and anti-obesity effects. Although existing evidence suggests the presence of AOSs in peripheral tissues after oral administration, whether AOSs permeate into the blood circulation remains unknown. Thus, we hypothesized that AOSs with low-molecular weight can permeate the human gastrointestinal tract. To test this hypothesis, the time course of absorption was examined by analyzing plasma samples before and 1, 2, and 4 h after ingestion. Analysis was performed using liquid chromatography/mass spectrometry after labeling with *p*-aminobenzoic ethyl ester. Our results showed that the plasma concentration of agarobiose (Abi) was higher than that of agarotetraose (Ate); however, agarohexaose was not detected. Additionally, plasma levels of Abi and Ate were proportional to the dose. These results suggest that permeation efficiency is dependent on the molecular weight and that the systemic absorption of Abi via the gastrointestinal tract is better than that of Ate. These findings will contribute to a better understanding of the bioactivity of orally administered AOSs in peripheral tissues.

## 1. Introduction

Polysaccharide agar is a galactan that forms a major cell wall component in red algal families such as Gelidiaceae and Gracilariaceae (1). Agar refers to a mixture of two polysaccharides, agarose and agaropectin. Agarose consists of alternating residues of (1 → 3)-linked β-d-galactose and (1 → 4)-linked 3,6-anhydro-α-l-galactose (2). Partial depolymerization of agarose via acid degradation leads to the hydrolysis of α-(1 → 3)-galactosidic bonds, resulting in the production of agaro-oligosaccharides (AOSs). AOSs are composed of repeating agarobiose (Abi) units, with 3,6-anhydro-α-l-galactose at the reducing end (3). AOSs exhibit promising applications in the food industry owing to their biological activities, possibly attributed to the presence of 3,6-anhydro-α-l-galactose at the reducing end (4).

AOSs are generally presumed to be indigestible by the human intestine owing to the lack of agarase production. There are many reports on the bioactivities of AOSs, such as anti-inflammatory (5, 6), anti-obesity (7), and prebiotic (8) effects, which can be explained by their local functioning in the gut or modulation of the gut microbiota, including the production of bioavailable microbial metabolites. Enoki et al. (9) reported that orally administered AOSs suppressed 12-*O*-tetradecanoylphorbol-13-acetate-induced tumor formation in mouse skin. The underlying mechanism might be explained by the changes in the gut microbiome; however, AOSs, especially low-molecular weight (MW) AOSs, which may be absorbed into the blood circulation, can have direct functions and exhibit their bioactivities in peripheral tissues.

The single oral administration test revealed that partially depolymerized shark chondroitin sulfate (CS) was detected in human plasma and urine (10). Pharmacokinetics parameters vary with the sulfation status, MW, or charge density of CS used on the experiment (11, 12). In an animal experiment using rats and dogs, orally administered CS was detected in the tissues, including the intestine, liver, kidneys, and joint cartilage (13).

In our previous study, AOS permeation from the intestine into blood circulation was examined *in vivo* and *in vitro* (14). Orally administered AOSs have been detected in rat plasma in a dose-dependent manner. The detection level in rat plasma was inversely correlated with the degree of polymerization, and Abi showed a higher plasma concentration than that of agarohexaose (Ahe), which has three repeating Abi units. Transport experiments in the Caco-2 cell culture model indicated that low-MW AOSs easily permeated the Caco-2 cell monolayers, and that the main transport route was the paracellular pathway. However, few clinical data are available on the absorption of AOSs from the gastrointestinal tract into the blood in humans.

Taking these findings into consideration, we hypothesized that low-MW AOSs can be absorbed into the human blood after oral administration. To test our hypothesis, we examined the plasma levels of AOSs after oral administration to healthy volunteers. Plasma samples were collected before and 1, 2, and 4 h after ingestion. To evaluate the absorption efficacy of various AOSs depending on their degree of polymerization, the time course for the absorption of Abi, agarotetraose (Ate), and Ahe was compared.

## 2. Methods

### 2.1. Chemicals

Acetonitrile, ethanol, and formic acid were purchased from FUJIFILM Wako Pure Chemical Corporation (Osaka, Japan). Agar was provided by Ina Food Industry Co., Ltd. (Nagano, Japan). The AOSs were obtained by the hydrolysis of agar under mild acidic conditions, according to the method described by Enoki et al. (4) with slight modification. Briefly, agar was dispersed in 0.1 N HCl and heated at 90°C for 30 min. After being cooled to 20°C–25°C, the solution was incubated with powdered activated carbon (Kirin Kyowa Foods Co., Ltd., Tokyo, Japan) for 60 min, filtered using a filter paper (Advantec Toyo Kaisha, Ltd., Tokyo, Japan) followed by a membrane filter (0.2 μm; Advantec Toyo Kaisha, Ltd.), and freeze-dried. The rate of Abi, Ate, and Ahe in AOSs were 30.2%, 29.1%, and 31.6%, respectively. For preparing calibration curves, Abi, Ate, and Ahe were isolated from the AOSs using size-exclusion chromatography according to the methodology described in our previous report (14). The MW of each AOS is provided in Table 1.

**Table 1:** Molecular weights of AOSs.

AOS	Molecular weight
Abi	324.3
Ate	630.5
Ahe	936.8

AOSs, agaro-oligosaccharides; Abi, agarobiose; Ate, agarotetraose; Ahe, agarohexaose.

### 2.2. Study design

This study was performed in accordance with the Declaration of Helsinki. All procedures were approved by the Ethics Committee of Tokyo Kasei University (certificate number R1-8). Written informed consent was obtained from each participant before enrollment. Negative effects have not been reported for AOS ingestion in human studies, and the safety of high AOS doses has been demonstrated in animal experiments (14). All volunteers were informed of our study objectives and the potential risks of AOS ingestion, such as diarrhea and abdominal pain, and the written consents were obtained from all participants after informed.

In total, 12 healthy volunteers (females) aged 21–26 years were enrolled in this study. The exclusion criteria were as follows: individuals taking daily supplements containing agar or AOSs; individuals allergic to agar or AOSs; and patients with conditions such as obesity, hyperlipidemia, hypertension, inflammatory bowel disease, or diabetes. In the first trial, 5 g AOSs were orally administered to all 12 participants. Four volunteers withdrew before starting the second trial because of personal reasons. In the second trial, after a washout period of 1 month, 2 g AOSs were orally administered to 8 participants. The dose was determined based on the no observed adverse effect level of galacto-oligosaccharide (15). For both the trials, 12 h of fasting preceded the oral administration, which entailed the ingestion of 200 mL water containing the AOSs.

No gastrointestinal problems were reported by any of the participants. Venous blood (10 mL) was collected from the cubital veins of the participants and stored in heparin-coated tubes immediately prior to and 1, 2, and 4 h after AOS ingestion. The blood samples were centrifuged at 1,000 × *g* for 10 min at 4°C, and the supernatants were collected. Ethanol was added to the isolated plasma samples to obtain a final concentration of 75% and vortexed. The samples were further centrifuged at 1,000 × *g* for 10 min at 4°C, and the supernatants were collected. For subsequent quantification, isotope-labeled internal standard glucose was used. This solution was added to 200 μL of the supernatant and evaporated to dryness.

### 2.3. Quantification of AOSs in human plasma

For high-sensitivity analysis, AOSs were labeled with *p*-aminobenzoic ethyl ester (ABEE) using an ABEE Labeling Kit (J-Chemical, Tokyo, Japan) according to our previous report (14), with slight modifications. Briefly, dried samples prepared from plasma were dissolved in 10 μL of distilled water, and 40 μL of the ABEE reagent solution was added. The mixture was incubated at 85°C for 1 h. After cooling to 15°C–20°C, 150 μL distilled water and chloroform were added, and the mixture was vigorously vortexed and centrifuged at 3,000 × *g* for 1 min at 20°C. The upper aqueous phase was filtered using TORAST Disc GLCTD-PTFE (13 mm, 0.22 μm, Shimadzu, Kyoto, Japan) and subjected to liquid chromatography/mass spectrometry (LC/MS) analysis. The monitored positive ions of ABEE-derivatized Abi, Ate, and Ahe showed *m*/*z* values of 474.2, 780.3, and 1086.4, which corresponded to protonated ions. The concentrations of Abi, Ate, and Ahe in the plasma were calculated using the standard curves of ABEE-Abi, ABEE-Ate, and ABEE-Ahe, respectively. The MS chromatograms, spectra, and standard curves were described in our previous study (14). The structures of ABEE-derivatized AOSs are shown in Supplementary Figure S1.

The LC/MS system consisted of an LC-20AD pump (Shimadzu) fitted with a DGU-20A 5R degassing unit (Shimadzu), an Inertsil octadecylsilyl column (150 × 2.1 mm, 5 μm, 100 Å; GL Sciences Inc., Tokyo, Japan), and an LCMS-2020 (Shimadzu) electrospray ionization MS device. The mobile phase consisted of (A) formic acid (0.1%) in water and (B) formic acid (0.1%) in acetonitrile, and the following gradient conditions were used: 0–5 min, 20% B; 5–10 min, 20–30% B; 10–15 min, 100% B; 15–20 min, 20% B. The flow rate was 0.4 mL/min, and the injection volume was 20 μL. The temperatures of the column oven and autosampler were set at 40°C and 10°C, respectively. Sample quantification was performed using selected ion monitoring in the positive-ion mode. The MS conditions were as follows: interface temperature, 350°C; desolvation line temperature, 250°C; heat block temperature, 200°C; nebulizer gas flow, 1.5 L/min; drying gas flow, 15 L/min; interface voltage, 4.5 kV; desolvation line voltage, 0 kV.

### 2.4. Statistical analysis

The area under the curve (AUC) for plasma concentration of each AOS was calculated using the trapezoidal formula. The plasma concentrations of Abi and Ate at each time point (1 h, 2 h, and 4 h after ingestion) were compared using the student’s paired *t*-test. The effect of each dose (5 g and 2 g) on the AUC of Abi and Ate was also evaluated using the student’s paired *t*-test. Statistical analysis was performed using Statcel 4 software (OMS Publishing Inc., Saitama, Japan). Data are presented as mean values ± standard deviation. A *p*-values of less than 0.05 was considered as statistically significant.

## 3. Results

**Figure 1:**
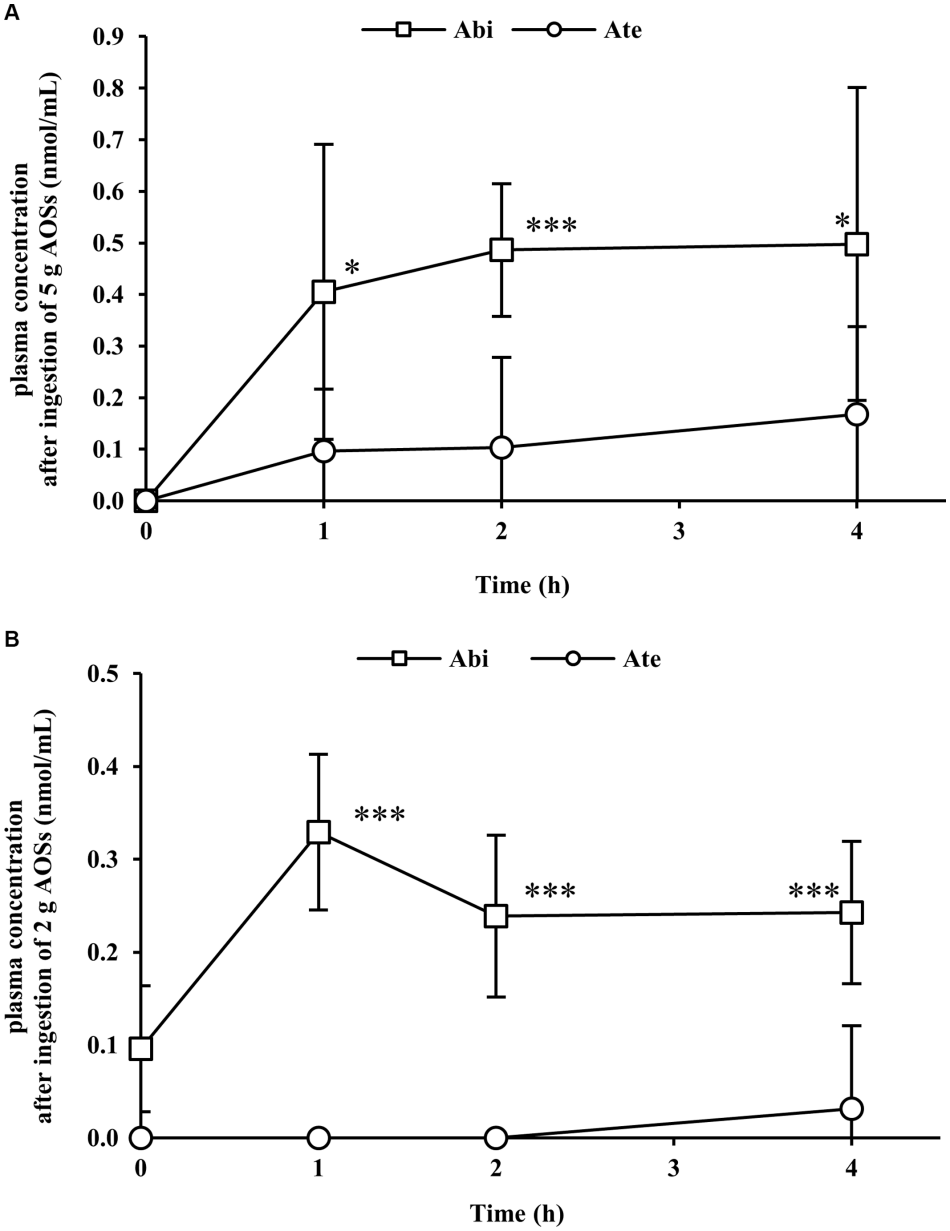
Plasma concentration–time profiles of agarobiose (Abi) and agarotetraose (Ate) after oral administration of 5 g **(A)** or 2 g **(B)** of agaro-oligosaccharides (AOSs). Venous blood was collected from the cubital veins of the participants before and 1, 2, and 4 h after ingestion of AOSs. Abi and Ate were detected in plasma samples via labeling with *p*-aminobenzoic ethyl ester. Abi displayed a higher concentration than Ate at each time point. The concentrations of Abi and Ate were dose-dependent. Data are presented as the mean ± standard deviation (*n* = 8). The plasma concentration of Abi was compared with that of Ate at each time point using the student’s paired *t*-test. The asterisk (^*^) and three asterisks (^***^) correspond to *p* < 0.05 and *p* < 0.001, respectively.

The absorption of AOSs with different MWs in the bloodstream was examined by the low-dose (2 g) and high-dose (5 g) trials. Administered AOSs comprised 30.2%, 29.1%, and 31.6% of Abi, Ate, and Ahe, respectively. The amounts of Abi, Ate, and Ahe in the 2 g and 5 g doses were 1.86, 0.92, and 0.68 mmol and 4.66, 2.31, and 1.69 mmol, respectively. The representative MS chromatograms of the volunteers included in the statistical analysis are shown in Supplementary Figure S2. The concentration-time profiles of the AOSs are shown in Figure 1. In the high-dose (5 g) trial, the plasma concentration of Abi and Ate were increased at 1 h after ingestion and were maintained at the same level or gradually increased up to 4 h after ingestion (Figure 1A). Abi was also detected in the low-dose (2 g) trial (Figure 1B). In contrast, plasma Ate was detected only in a 4 h sample obtained from one of the eight participants. The plasma concentration of Abi at all time points (1 h, 2 h, and 4 h) was significantly higher than that of Ate in both trials. The plasma Ahe level was below the detection limit in both the trials. These results indicate that the permeation of AOSs is inversely correlated with their MWs.

**Figure 2:**
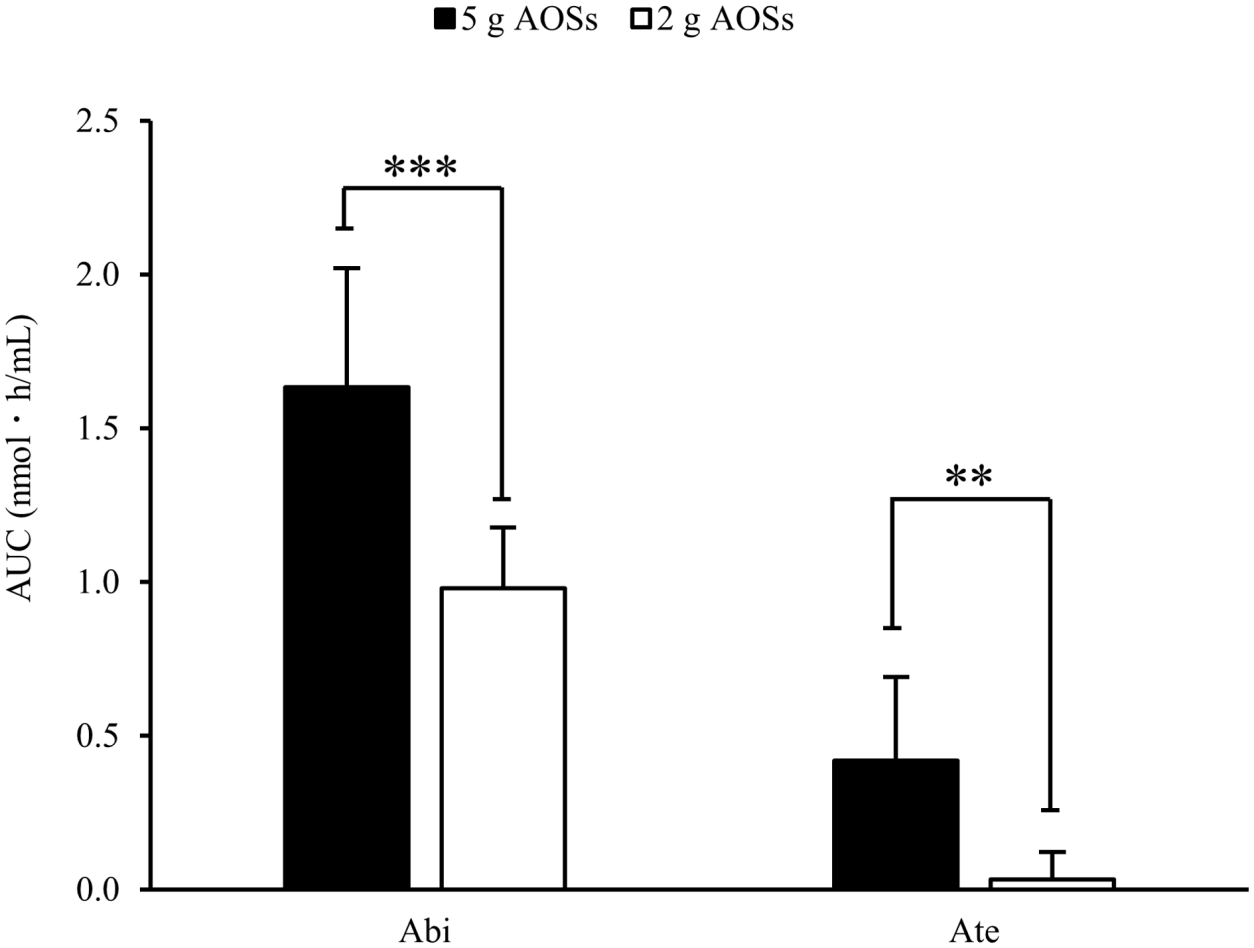
Area under the curve (AUC) of Abi and Ate after oral administration of AOSs. AUC for the plasma concentration was calculated using the trapezoidal formula. The effect of each dose on the AUCs of Abi and Ate was evaluated using the student’s paired *t*-test. Data are presented as the mean ± standard deviation (*n* = 8). The two asterisks (^**^) and three asterisks (^***^) correspond to *p* < 0.01 and *p* < 0.001, respectively.

The AUC for the plasma concentration of Abi and Ate was also examined (Figure 2). After the ingestion of 5 g AOSs, the AUC value of plasma Abi was 1.59 nmol‧h/mL (range: 1.17–2.35 nmol‧h/mL) (Figure 2). With low-dose AOSs (2 g), the AUC value of plasma Abi was 0.96 nmol‧h/mL (range: 0.84–1.26 nmol‧h/mL). The plasma AUC of Ate was 0.41 nmol‧h/mL (range: 0.00–0.88 nmol‧h/mL) in the high-dose trial and 0.03 nmol‧h/mL (range: 0.00–0.25 nmol‧h/mL) in the low-dose trial. The AUC in the high-dose trial was significantly larger than that in the low-dose trial for both Abi and Ate. These results suggest that the plasma levels of AOSs are dose-proportional, independent of the MW.

## 4. Discussion

In this study, pharmacokinetics of AOSs in human plasma after oral ingestion were examined. The protocol was determined based on our previous report. Pharmacokinetic study in rats revealed that the plasma levels of AOSs are maximum at 4 h and significantly decrease 8 h after oral administration (14). Therefore, time points selected for blood collection were before AOS administration (0 h) and 1, 2, and 4 h after AOS administration.

In the pre-ingestion sample of the low-dose trial, an unidentified peak was observed at the same selected ion monitoring channel as ABEE-derivatized Abi. However, it was unlikely to represent ABEE-derivatized Abi given the absence of endogenous AOSs in humans and the 12 h of fasting before the oral administration of the AOSs. Therefore, we regarded the unidentified peak as the baseline and subtracted it from the AUC values.

The amount of detected AOSs in the plasma increased after oral administration, with an inverse correlation between the plasma detection levels and the MW of the AOSs. In the high-dose (5 g) trial, the AUC values of plasma Abi and Ate were 1.59 and 0.41 nmol‧h/mL, respectively, which means that permeation efficacy of Abi was almost four times higher than that of Ate. Since Ate has nearly two times higher MW than Abi, the amount of Ate contained in AOSs is halved compared to Abi. Therefore, the difference between the AUC values of Abi and Ate was not fully explained by their concentration. Low-MW AOSs could permeate more efficiently compared with high-MW AOSs.

Considering the absence of agarase in the mammalian gastrointestinal tract, Abi and Ate remained intact until their elimination in urine. Similarly, human milk oligosaccharides also exist in human plasma for considerable time (16). Although the time course of plasma concentration is unclear, human milk oligosaccharides were detected in human plasma and were found in infants’ urine with a peak time of 8–12 h. Hence, it is possible that the plasma concentrations of Abi and Ate beyond 4 h were also maintained at certain levels.

A recent study evaluated the pharmacokinetic parameters of orally administered CS in human plasma (17). In this study, the absorption rate of CS was higher than that of AOSs. A single dose of 2,400 mg CS with a MW of 5,120 Da increased the plasma CS concentration from baseline to 48 h. The intake-standardized AUC value was more than eight times higher than that of Abi and sixteen times higher than that of Ate. In addition, the intake-standardized maximum plasma concentration was more than 14 and 34 times higher than that of Abi and Ate, respectively. According to a study using Caco-2 cell monolayers, both CS (18) and AOSs (14) across Caco-2 cell monolayers via the paracellular pathway. These findings are in good agreements with previous reports in which the transport of oligosaccharides occurred via the paracellular pathway (19). The permeability of low-MW hyaluronan increased inversely with its molecular size (20), which is also consistent with our findings. However, the underlying mechanism responsible for the different rates of absorption of CS and AOSs still remains unclear.

High-MW oligosaccharides might be utilized by the gut microbiota, but they are generally regarded to be poorly absorbed through the intestinal barrier. In fact, unlike Abi, Ahe was not detected in human plasma after oral administration in this study. Low-MW AOSs absorbed in the blood circulation might exhibit their biological effects in peripheral tissues. In other words, the biological activities of AOSs are mainly caused by Abi because of its higher concentration in blood. Enoki et al. (4) reported that AOSs inhibited the production of interleukin-1β and interleukin-6 in mouse RAW264.7 macrophages, demonstrating the anti-inflammatory activity of AOSs. However, it is still unclear whether low-MW AOSs transferred to the blood can interact with macrophages located in peripheral tissues and exhibit their anti-inflammatory activity. Orally administered raffinose oligosaccharide can be detected in rat plasma (21). Furthermore, dietary raffinose reduced the number of eosinophils in the broncho-alveolar lavage fluid and alleviate allergic inflammation in the lung tissue of ovalbumin-sensitize rats. The ameliorative action of dietary raffinose was observed in neomycin-treated rats, suggesting a post-absorptive action of raffinose in lung tissues without a prebiotic action. Therefore, further *in vivo* investigation is necessary to determine the accumulation of AOSs in peripheral tissues after oral administration.

We demonstrated that single-dose orally administered AOSs could be detected as Abi and Ate in human plasma. However, the ingestion of repeated AOS doses may potentially improve their absorption rate. For example, after 28 days of oral administration of coenzyme Q10, its plasma concentration was higher than that after a single administration, indicating an increase in bioavailability with repeated administration (22). Future studies are needed to investigate the influence of continuous AOS supplementation on bioavailability.

In conclusion, this study indicated that Abi and Ate were absorbed into human plasma following oral ingestion, with concentration levels maintained for the experimental period of 4 h. The plasma levels of AOSs were dose-dependent and inversely correlated with their MW. Abi showed the highest concentration in human plasma, in accordance with our previous animal study; in contrast, Ahe was not detected in any plasma sample. These results imply that low-MW AOSs may be localized in peripheral human tissues where they can exert their biological effects.

## Data availability statement

The original contributions presented in the study are included in the article/Supplementary material, further inquiries can be directed to the corresponding authors.

## Ethics statement

The studies involving humans were approved by the Ethics Committee of Tokyo Kasei University. The studies were conducted in accordance with the local legislation and institutional requirements. The participants provided their written informed consent to participate in this study.

## Author contributions

IS: Conceptualization, Data curation, Investigation, Writing – original draft. YI: Data curation, Investigation, Writing – original draft. KK: Conceptualization, Writing – review & editing. YS: Conceptualization, Writing – review & editing. SK: Conceptualization, Writing – review & editing.

## Conflict of interest

IS and KK are employees of Ina Food Industry Co., Ltd.

The remaining authors declare that the research was conducted in the absence of any commercial or financial relationships that could be construed as a potential conflict of interest.

## Publisher’s note

All claims expressed in this article are solely those of the authors and do not necessarily represent those of their affiliated organizations, or those of the publisher, the editors and the reviewers. Any product that may be evaluated in this article, or claim that may be made by its manufacturer, is not guaranteed or endorsed by the publisher.

## Abbreviations

ABEE: *p*-aminobenzoic ethyl ester
Abi: agarobiose
Ahe: agarohexaose
AOS: agaro-oligosaccharides
Ate: agarotetraose
AUC: area under the curve
CS: chondroitin sulfate
LC/MS: liquid chromatography/mass spectrometry
MW: molecular weight
