# Supplementary material for: Orally administered low-molecular weight agaro-oligosaccharides are absorbed into the plasma of healthy humans
Source: Front Nutr. 2023 Sep 25;10:1273328. doi: 10.3389/fnut.2023.1273328 (PMC10560721; doi:10.3389/fnut.2023.1273328)
Supplement: Supplementary file 1 [file Data_Sheet_1.docx]

Supplementary Material

**Supplementary Method.** Preparation of AOSs and isolation of Abi, Ate, and Ahe

Agar was suspended in 0.1 N HCl to 10% (w/v) and heated at 90°C for 30 min. After being cooled to room temperature, powdered activated carbon was added and incubated for 60 min. Powdered activated carbon was filtered, and the AOSs solution was freeze-dried. The yield was 85.1%.

The oligosaccharides of different sizes were isolated from the AOSs through size exclusion chromatography using a flash column (15 mm × 170 cm) packed with the Toyopearl HW-40S (Tosoh, Tokyo, Japan) stationary phase at a mobile phase (distilled water) flow rate of 0.1 mL/min to fractionate the AOSs with different molecular weights. Each fraction obtained was analyzed with an HPLC-refractive index detector (RID-10A, Shimazu, Kyoto, Japan) system. Each fraction was separated on a TSKgel G2500 PWXL (300 × 7.8 mm, 7.0 μm; Showa Denko K.K., Tokyo, Japan). The mobile phase was distilled water and sent using an LC-10AT pump (Shimazu). The fractions were collected and freeze-dried.

The composition of AOSs was determined by liquid chromatography/mass spectrometry (LC/MS) analysis using standards of Abi, Ate, and Ahe. AOSs comprised 30.2%, 29.1%, and 31.6% of Abi, Ate, and Ahe, respectively.


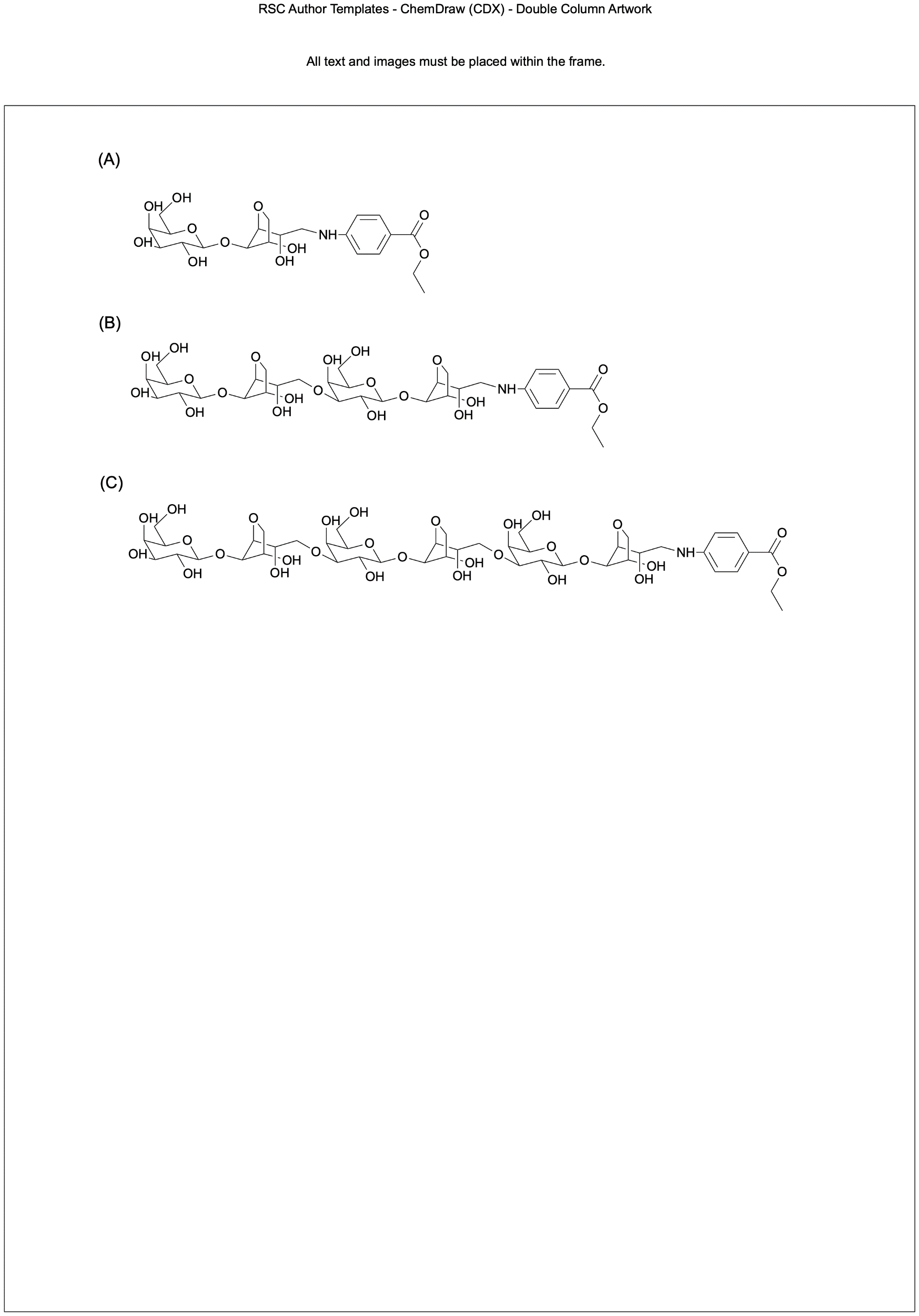


**Supplementary Figure 1.** Structures of *p*-aminobenzoic ethyl ester-derivatized agaro-oligosaccharides (ABEE-AOSs). AOSs were converted to ABEE at their reducing ends, producing ABEE-Abi **(A)**, ABEE-Ate **(B)**, and ABEE-Ahe **(C)**.


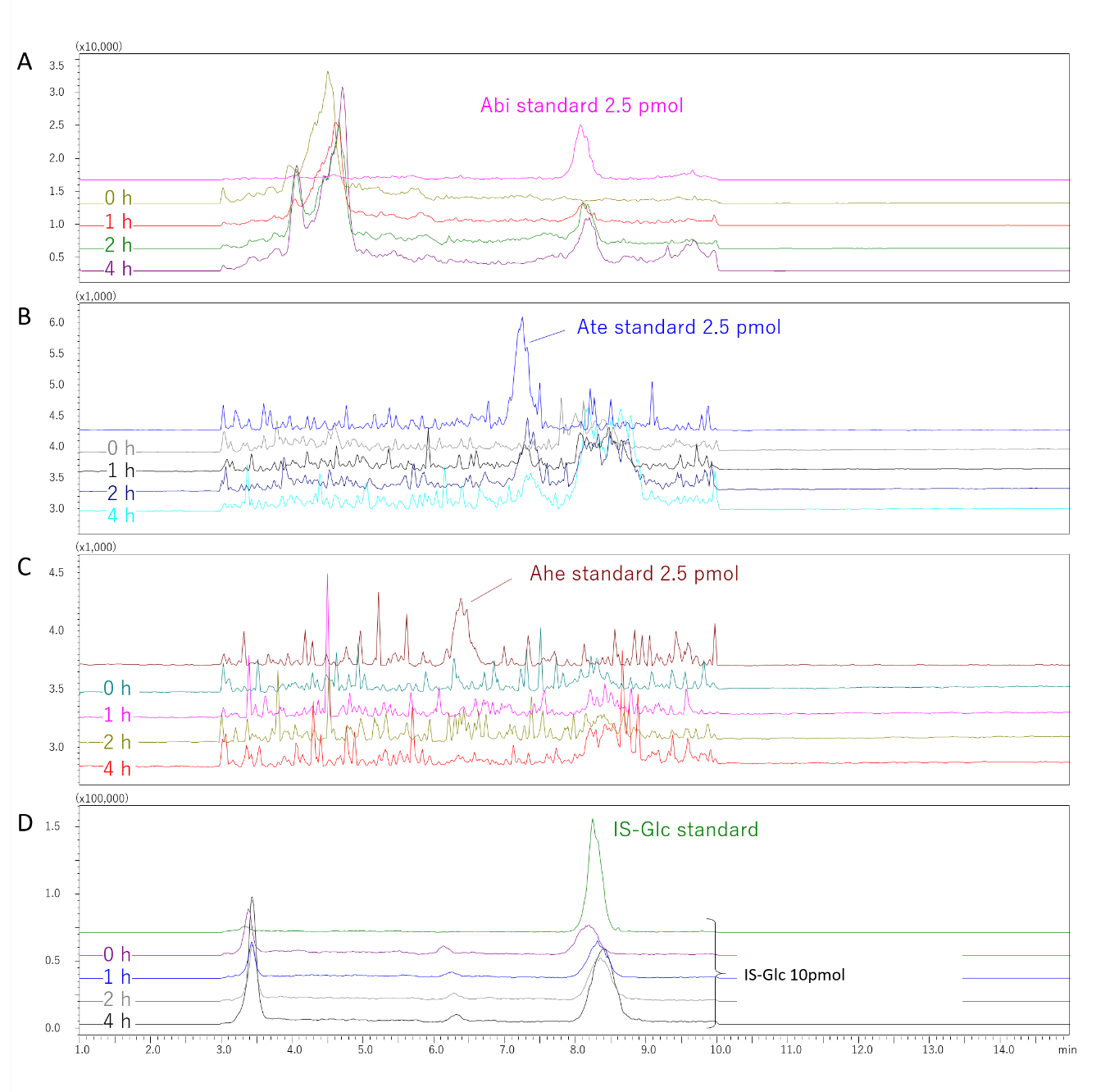


**Supplementary Figure 2.** MS chromatogram of Abi, Ate, Ahe, and IS-Glc obtained from the plasma sample of volunteer No.1.

The plasma sample of volunteer No.1 was applied to LC/MS. MS chromatogram of standard sample of Abi (A), Ate (B), Ahe (C), and IS-Glc (D) was shown with MS chromatogram obtained from the plasma sample.
